# Isolated parachute mitral valve in a 29 years old female; a case report

**DOI:** 10.15171/jcvtr.2016.08

**Published:** 2016-03-15

**Authors:** Mehrnoush Toufan, Seyed Sajjad Mahmoudi

**Affiliations:** ^1^Cardiovascular Research Center, Tabriz University of Medical Sciences, Tabriz, Iran; ^2^Department of Cardiology, Cardiovascular Research Center, Tabriz University of Medical Sciences, Tabriz, Iran

**Keywords:** Isolated Parachute Mitral Valve, Shone’s Complex, Mitral Stenosis

## Abstract

A 29-year old female patient was referred to our hospital for evaluation of dyspnea NYHA class I which begun from several months ago. The only abnormal sign found on physical examination was a grade 2/6 systolic murmur at the apex position without radiation. Echocardiography revealed normal left and right ventricular sizes and systolic function, and only one papillary muscle in left ventricular (LV) cavity which all chordae tendineae inserted into that muscle. The mitral valve orifice was eccentrically located at the lateral side with mild to moderate mitral regurgitation but without significant mitral stenosis. No other congenital heart anomalies were identified. Thus, the final diagnosis was isolated parachute mitral valve (IPMV). She was one of the very rare IPMV cases have ever been reported in adults

## Introduction

The mitral valve is a functional complex that relies on normal morphology, geometrical relations and function of all its constituents: annulus, leaflets, the subvalvular apparatus including chordae tendineae and papillary muscles.^1^ In a parachute mitral valve (PMV), only one papillary muscle exists and all chordae tendineae which are usually shorter and thicker than normal type, inserted into this single muscle. This condition restricts the motion of leaflets and obstructs the blood flow into the left ventricle during diastole.^2^ In the current study, we report a rare case of isolated PMV (IPMV) who has remained undetected till 29 years old.

## Case Report

A 29-year-old female patient was referred in August 2013 to the echocardiography unit of Tabriz Madani Heart Center, for further evaluation of dyspnea NYHA class I, which begun since 3 months ago. Her past medical history was negative. Her general growth and development were in normal condition and there was no dysmorphic features. She had normal HR (82 bpm) and normal blood pressure (BP) (110/70 mmHg) in both upper and lower limbs. The heart sounds were also regular but there was a grade 2/6 systolic murmur at the apex position without radiation. Additional physical examination and other assessments including laboratory tests, electrocardiogram and chest x-ray were normal.

**Figure 1:**
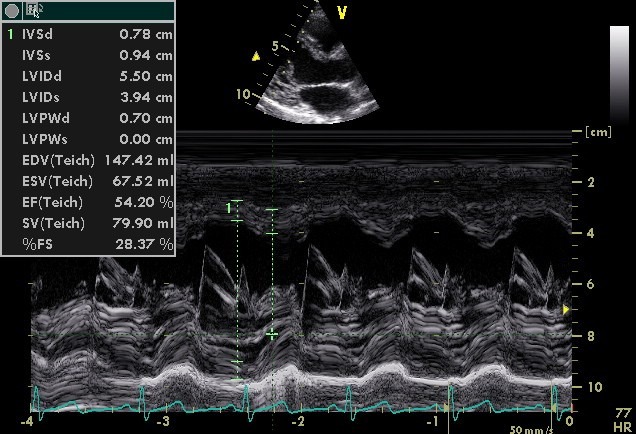
M-mode parasternal long axis view shows thickened chordae meshwork in LV cavity.

**Figure 2:**
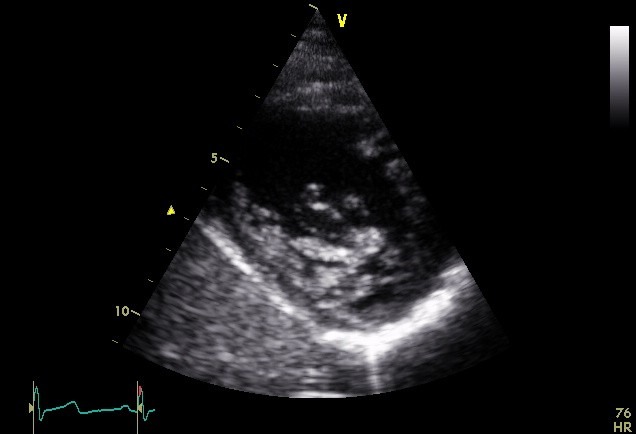
Parasternal short axis view on mid- papillary level shows a single postromedial muscle.

**Figure 3:**
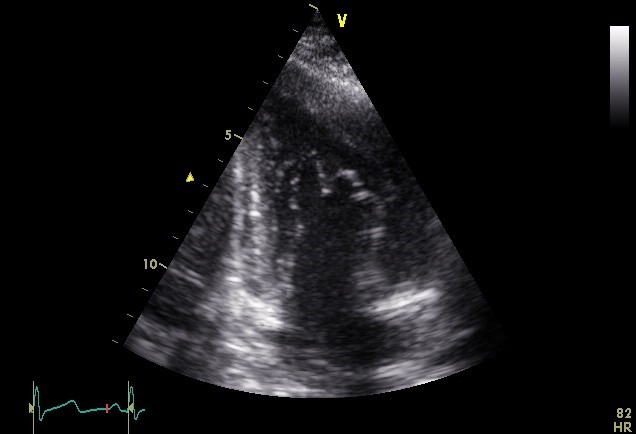
Apical two chamber view demonstrates typical parachute appearance of mitral chordae

Transthoracic echocardiography revealed normal left ventricular size (LVEDD: 55 mm), enlarged left atrium (LA volume index: 45 ml/m^2^), normal right sided chambers and systolic function was preserved in both sides of the heart (LVEF: 55%). Only one papillary muscle was seen in medial side in LV short-axis view on mid-papillary level. All thickened and elongated chordae converged into single papillary muscle, seen in LV long-axis view. Also, dysplastic chordae tendineae and thickened mitral valve leaflets were seen in four-chamber view (Figures 1-3) (Supplementary 1). The mitral valve orifice was eccentrically located at the lateral side with mild to moderate mitral regurgitation but without significant mitral stenosis. There were no aortic coarctation, thickening of the mitral annulus or supravalvular mitral membrane or any other defects. Thus, we diagnosed it as an IPMV.

The patient was just prescribed low dose diuretic (Furosemide 20 mg daily) and advised to annually follow up assessment that is continuing till now, for about two years without new changes in clinical or echocardiographic findings.

## Discussion

PMV is an unusual congenital defect of the mitral valve most frequently accompanied by other deformities of the left heart such as aortic coarctation, valvular and subvalvular aortic stenosis and supravalvular mitral membrane, generally named as the complex of Shone.^2^ Although, extensive hemodynamic effects lead to diagnosis almost during infancy and childhood and remaining undiagnosed till adulthood is very uncommon, however a number of incomplete forms have been detected in adult patients.^3,4^ Adults with PMV experience modest defects that do not need for echocardiography and may lead to under diagnosis of the anomaly.

To best of our knowledge and according to the systematic review by Hakim et al,^4^ only nine cases of adults PMV have ever been reported till 2010. Of them five patients had isolated PMV, but one subject was diagnosed after death on autopsy who presented by sudden death,^5^ one patient was referred due to uncontrolled hypertension^6^ and three of them were suffered from progressive dyspnea as well as our patient. In recent years, two new isolated PMV cases has been reported in two 40- and 73-year-old females accompanying moderate to severe mitral regurgitation and moderate mitral stenosis, respectively.^7,8^

The complexity and severity of anomalies may be the determinant of the wide range of symptoms; however dyspnea is the most frequent symptom of adult PMV.^9-11^ Because opening of the mitral valve is limited, true PMV is highly associated with mitral stenosis. Mitral regurgitation occurs less commonly but must be carefully followed because of its progressive evolution.^3,12^

Conservative surgical treatment may consist of either chordal fenestration or papillary muscle splitting, with or without commissurotomy.^13^ The prognosis is usually poor and unsatisfactory outcomes have been reported from surgical repair. Though, surgical *correction* is typically chosen in cases of *obstructive* PMV, due to absence of significant mitral stenosis or regurgitation in echocardiographic evaluation, our patient underwent medical therapy and annual surveillance. Now, after two years follow-up, she has no new obvious change in clinical and echocardiographic findings.

## Ethical issues

This study was approved by the committee of ethics of Tabriz University of medical sciences.

## Competing interests

Authors declare no conflict of interest in this study.

## Supplementary files

 Supplementary 1 consists of video file.
